# High Preponderance of *BRAF* V600E Mutation in Papillary Thyroid Carcinoma Among Filipinos: A Clinicopathologic Study

**DOI:** 10.1200/JGO.18.00085

**Published:** 2019-01-29

**Authors:** Gerard Anthony M. Espiritu, Joemarie T. Malana, Arlie Jean Grace V. Dumasis, Daphne C. Ang

**Affiliations:** **Gerard Anthony M. Espiritu**, **Joemarie T. Malana**, and **Daphne C. Ang**, Makati Medical Center, Makati; and **Arlie Jean Grace V. Dumasis** and **Daphne C. Ang**, St Luke’s Medical Center, Taguig, Philippines.

## Abstract

**Purpose:**

*BRAF* mutation in papillary thyroid carcinoma (PTC) is associated with an aggressive phenotype, with varying incidence. We evaluated the prevalence of *BRAF* mutations in PTC among Filipino patients and their correlation with clinicopathologic characteristics.

**Patients and Methods:**

Clinicopathologic data were retrieved from 64 sequential patients who underwent thyroidectomy from June 2016 to December 2016. *BRAF* mutation testing was performed using Sanger sequencing.

**Results:**

Eighteen (28%) of 64 patients were diagnosed with PTC; 12 (70.59%) of 17 harbored a *BRAF* V600E mutation (no amplification in one patient). Demographics of patients with PTC were as follows: 13 women and five men, with median age of 46 years (range, 25 to 74 years). Fourteen patients had conventional subtype PTC; two, follicular variant; one, oncocytic variant; and one, tall-cell features. Tumor size ranged from 0.8 to 7.0 cm (median, 2.4 cm); extrathyroidal extension was present in seven (38.9%) of 18 patients, multifocality in six (33.33%) of eight, and lymph node involvement in eight (44.4%) of 18. Significant association between presence of a *BRAF* mutation and presence of extrathyroidal extension or lymph node involvement was not determined due to the limited sample size.

**Conclusion:**

The high preponderance of *BRAF* mutation (70.59%) suggests some correlation with the previously reported lower 5-year survival among Filipinos. This warrants further investigation in a larger-cohort prospective study.

## INTRODUCTION

The most common endocrine neoplasm is thyroid carcinoma, with 80% to 85% cases classified as papillary thyroid carcinoma (PTC).^1^ Thyroidectomy can cure well-differentiated thyroid carcinoma, especially if discovered before formation of local or distant metastases.^2,3^ However, the 5-year survival rate was reported to be lower in a study among Filipinos with PTC in Hawaii.^4^

Oncogenic conversion of cell signaling pathways involving mitogen-activated protein kinase (MAPK) and phosphatidylinositol 3-kinase/protein kinase B (AKT) has been identified in the pathogenesis of thyroid cancer.^1^ *BRAF* (MAP3K), *RAS* (small GTP-binding protein), and *RET* (receptor tyrosine kinase) are drivers of the MAPK signaling cascade that have been of particular interest over the past 13 years.^5,6^ Incidence of *BRAF* mutations varies according to histologic subtype and geographic location, with rates ranging from 28% to 83%.^3,4,7,8^

Molecular profiling of *BRAF* mutations has been extensively studied in Western countries, with varying results. In 2004, a study in Milan, Italy, evaluated 60 cases of PTC for mutations in *BRAF*, *RAS*, *RET*, and *NTRK1* genes. *BRAF* mutations were investigated in exons 11 and 15.^5^ Thymine-to-adenine transversion at nucleotide 1,796 was demonstrated in 19 (31.7%) of 60 PTCs, leading to valine-to-glutamate substitution at residue 599. Different *BRAF* mutations were also found in two separate samples (G-to-A transition at nucleotide 1,365 in exon 11 and G-to-A transition in intron 11). A similar study was performed in 2005^2^ at the University of Michigan and University of Cincinnati Medical Center, which analyzed 51 cases of PTC and found a relative frequency of *BRAF* mutations of 61.0%. Tumors with *BRAF* mutations were either tall-cell variant or classic type. Another study in Naples, Italy, performed in 2006,^9^ identified *BRAF* mutations in PTC by mutant allele–specific polymerase chain reaction amplification. In contrast to earlier studies, *BRAF* mutations in this study were found to occur almost equally in both classic PTC (45.2%) and follicular PTC (41.7%). This nonconcordance was attributed to the different platforms used for determining mutations. In a larger-cohort study (500 consecutive cases of PTC), a *BRAF* V600E transversion was found in 214 (42.8%) of 500 cases of PTC.^3^

Molecular profiling of PTC has been of recent interest in Asia. In 2014, a study at Seoul St Mary’s Hospital analyzed *BRAF* V600E alleles in predicting PTC progression, and a significant association between presence of extrathryoidal extension, absence of chronic lymphocytic thyroiditis, and increase in tumor size and presence of *BRAF* V600E mutation was demonstrated.^7^

Among Filipinos, varying results in occurrence of a *BRAF* mutation were found. In 2011, a study in Hawaii^4^ showed a high incidence (83.8%) of *BRAF* mutations among its Filipino population. A study in the Philippines, however, showed a lower prevalence (38.6%) of *BRAF* mutations among Filipinos.^10^ Given the varying incidence of *BRAF* mutations, our study is concerned with a mutational analysis (exon 15 of the *BRAF* gene) among Filipinos with PTC and seeks to establish the diagnostic and prognostic significance of this mutation.

## PATIENTS AND METHODS

This study was conducted in accordance with the ethical principles based on the Declaration of Helsinki and the National Guidelines for Biomedical Research of the National Ethics Committee of the Philippines. Approval by the institutional review board of Makati Medical Center was obtained.

Histopathologic reports of thyroidectomy specimens from July 2016 to December 2016 were reviewed using the laboratory information system of the Anatomic Pathology Section of the Department of Pathology and Laboratories at Makati Medical Center. All thyroidectomy specimens diagnosed with PTC and/or papillary thyroid microcarcinoma were included in the study. Specimens with the same diagnosis but obtained by procedures other than thyroidectomy were excluded from the study.

Stored slides of the included cases stained with hematoxylin and eosin were reviewed, retrieved, and classified according to the WHO classification of PTC.^11^ The corresponding paraffin blocks of these cases were also retrieved, and six to 10 microsections were obtained using a microtome set at a 4-µm thickness. Macrodissection was performed for specimens with a lesion less than 1.0 cm in size.

DNA was extracted using the QIAamp DNA Mini Protocol for DNA Purification from Tissues (Qiagen, Hilden, Germany). Yield was determined from the concentration of DNA in the eluate through measurement of absorbance at 260 nm using Thermo Scientific NanoDrop 2000 (Thermo Fisher Scientific, Waltham, MA).

*BRAF* exon 15 was amplified using the following primers: forward primer 5′-TGCTTGCTCTGATAGGA-3′ and reverse primer 5′-GGCCAAAAATTTAATCAGTGG-3′. Sanger sequencing was performed using the ABI3500 Genetic Analyzer (Thermo Fisher Scientific).

Measures of central tendency and frequency distribution were determined.

## RESULTS

A total of 64 thyroidectomy cases from July 2016 to December 2016 were reviewed. Eighteen of 64 patients who underwent thyroidectomy were included in the study; 17 of these 18 patients were diagnosed with PTC, and one of 18 was diagnosed with papillary thyroid microcarcinoma. Patient demographics were as follows: five men (25%) and 13 women (75%; female-to-male ratio, 2.6), with an age range of 25 to 74 years (median age, 46 years; Table 1).

**Table 1:**
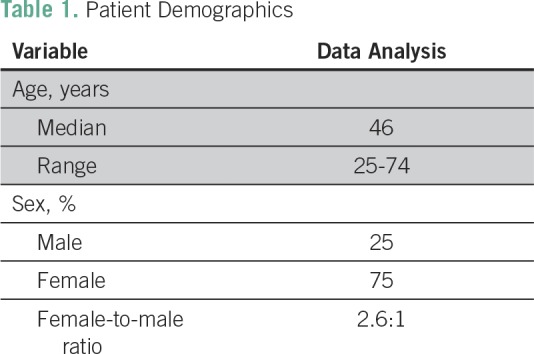
Patient Demographics

**Fig 1:**
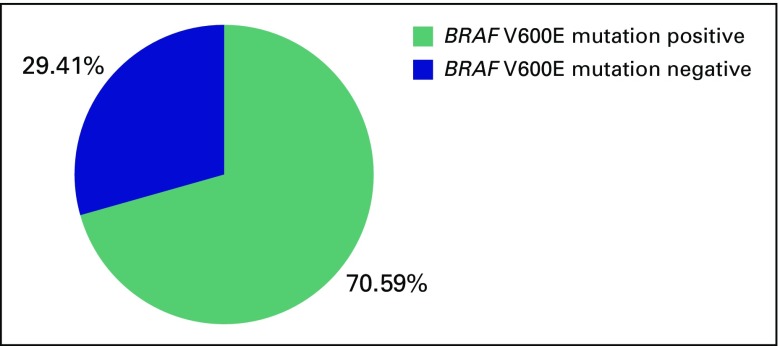
Percent distribution of *BRAF* V600E mutation.

Twelve (70.59%) of 17 patients with PTC harbored a *BRAF* V600E mutation (Fig 1), and one patient (classified as having tall-cell variant of PTC) failed to amplify, which may be because of degenerated DNA molecules.

Regarding PTC histologic subtype, 14 patients had conventional variant (77.78%), two had follicular variant (11.10%), one had oncocytic variant (5.56%), and one had tall-cell variant (5.56%; Table 2). Occurrences of *BRAF* V600E mutation among the histologic subtypes were as follows: 11 of 14 with conventional PTC, one of two with follicular variant, and one of one with oncocytic variant (Table 3). Tumor size ranged from 0.8 to 7.0 cm, with a median size of 2.4 cm. Lobe involvement was as follows: six patients (33.33%) with multicentric involvement, five (27.78%) with right lobe, six (33.33%) with left lobe, and one (5.56%) with isthmus (Table 2).

**Table 2:**
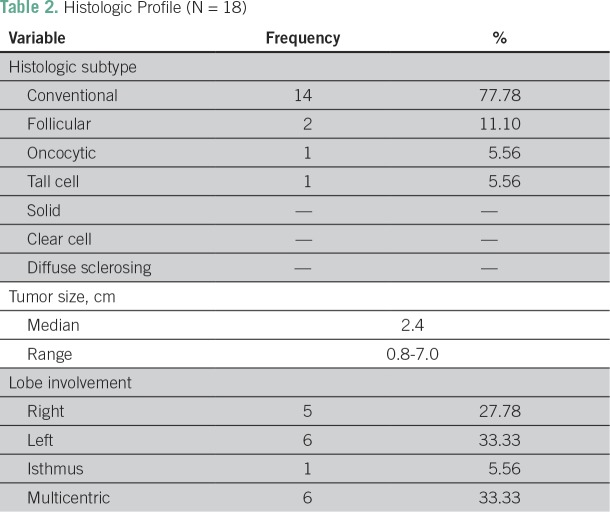
Histologic Profile (N = 18)

**Table 3:**
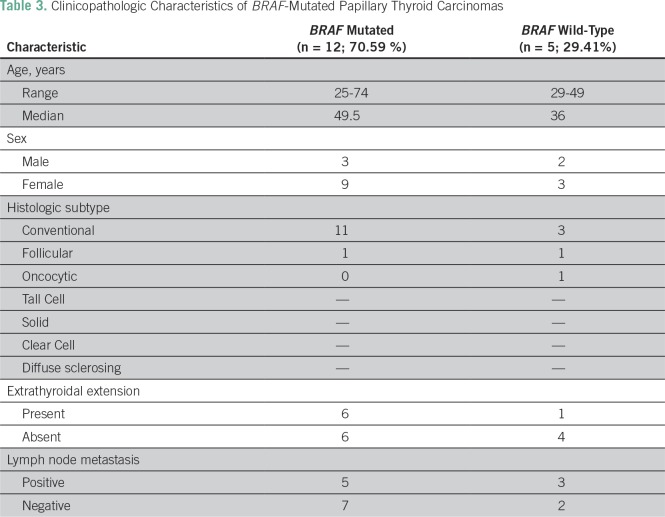
Clinicopathologic Characteristics of *BRAF*-Mutated Papillary Thyroid Carcinomas

Six (85.71%) of seven patients with PTC with extrathyroidal extension expressed a *BRAF* V600E mutation, and six (60%) of 10 patients with PTC without extrathyroidal extension also expressed a *BRAF* V600E mutation (Table 3)..

Five (62.5%) of eight patients with PTC with lymph node metastasis expressed a *BRAF* V600E mutation, while seven (77.78%) of nine PTC without lymph node metastasis harbored the mutation (Table 3). 

## DISCUSSION

Patient demographics in our study (median age, 46 years; female predominance), as listed in Table 1, were maintained when compared with the studies in Korea (median age, 46 years; female-to-male ratio, 3.2:1) and Hawaii (mean age, 47 years; female-to-male ratio, 3.1:1) and another Philippine study (mean age, 45 years; female predominance).^4,6,7,10,12^ This suggests homogeneity in the population characteristics.

The histologic subtypes of the PTC cases in our study (Table 2) are similar to those in the studies of Gertz et al^3^ and Navarro-Locsin et al,^10^ in which the conventional type of PTC predominated. Interestingly, the largest tumor size in our study (7.0 cm; median, 2.4 cm) is higher than those in the studies in the United States (2.8 cm; median, 1.7 cm) and Korea (4.6 cm; median, 0.7 cm).^3,7^ This may be attributed to the culture of Filipinos to tolerate clinical symptoms, resulting in late professional consult, or in some cases to poor access to health care services, or perhaps to a more aggressive behavior of PTC among Filipinos.

The high percent distribution (70.59%) of *BRAF* V600E mutation among patients with PTC in our study (Table 3) parallels those of studies conducted in Korea (incidence rate, 75.8% to 82.3%), China (incidence rate, 62.7%), Hawaii (83.8%), Poland (72.4%), and the United States (61.0% to 77.0%).^6-8,12^ These findings are consistent with the increasing trends in the prevalence of the mutation in PTC as years pass by.^8^ No specific reason for this increase has been identified, but the positive correlation may be due to advances in the test methods.^9^ Another possible cause of the increasing trend may be due to defective mismatch repair activity associated with *BRAF* V600E mutations.^13^ This defective mismatch repair was attributed to excessive oxidative stress during thyroid hormone synthesis, however, conflicting results were found in the correlation of iodine (component in thyroid hormone synthesis) levels and *BRAF* mutations.^8^ This area is worth investigating further.

Our study slightly differs from the other local (Philippine) study (38.6%).^10^ This may be a result of the difference in test methods and sensitivities.

General association between histologic subtypes and incidence of *BRAF* V600E mutation could not be evaluated because of the limited sample size of the other subtypes. The most predominant subtype of PTC in our study was the conventional type (Table 2). Among patients with conventional PTC, 78.57% (11 of 14; Table 3) harbored the mutation. This is comparable to the studies from Korea (82.3%) and Hawaii (83.8%), in which evaluation of an association between conventional PTC and *BRAF* mutation was performed.^4,7,14^

*BRAF* V600E mutation has been linked to a more aggressive phenotype of PTC in terms of extrathyroidal extension, nodal metastasis, and absence of capsule.^3^ In our study, frequency of *BRAF* V600E mutation was slightly higher in patients with extrathyroidal extension (85.71%) when compared with those without extrathyroidal extension (60%), but statistical significance cannot be determined due to the small sample size. This difference may be a result of the varying molecular subtypes within the *BRAF* V600E–mutated PTC group.^14^ The Cancer Genome Atlas study on *BRAF*-mutated PTC demonstrated that this is a heterogeneous group and identified at least four different molecular subtypes with distinct association with invasion and metastasis.^14^

Our study is limited by the small sample size, short duration, and single center retrospective design. A prospective study in a larger, multicenter study is warranted.
